# Greener Synthesis of Poly(LIM-*co*-DVB-*co*-AMPS): A Sustainable Approach to Methylene Blue Removal

**DOI:** 10.1021/acsomega.4c00354

**Published:** 2024-12-12

**Authors:** Aslı Erdem Yayayürük, Nevin Çankaya, Onur Yayayürük

**Affiliations:** † Faculty of Science, Department of Chemistry, Ege University, İzmir 35100, Turkey; ‡ Vocational School of Health Services-Oral and Dental Health Department, Uşak University, Uşak 64200, Turkey

## Abstract

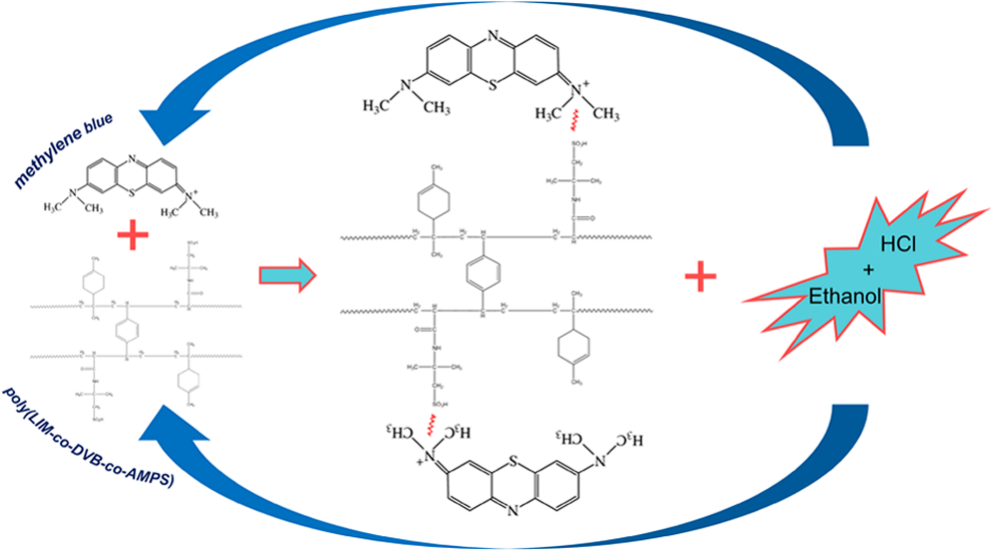

A novel environmentally friendly adsorbent, poly(limonene-*co*-divinylbenzene-*co*-2-acrylamido-2-methyl-1-propanesulfonic acid, LIM-*co*-DVB-*co*-AMPS), was synthesized and applied for the adsorption of methylene blue from aqueous solutions in this study. The structure, morphology, and thermal stability of the green adsorbent were determined by the FTIR, SEM, TGA/DTA/DTG, and BET techniques, ζ potential, and elemental analysis. The efficiency of the adsorption process was improved with respect to several experimental conditions, viz., adsorbent dose, pH, and contact time. The adsorption process was found to fit very well with the Langmuir isotherm and the pseudo-second-order model. Benefiting from the higher number of surface sites, porous structure, and good surface area, poly(LIM-*co*-DVB-*co*-AMPS) particles exhibited a superior adsorption performance for MB with a Langmuir adsorption capacity of 98 mg g^–1^. The selectivity of the sorbent does not depend on the coexisting ions, and the sorbent is applicable in complex matrixes in the presence of these ions. The elution process was employed using ethanol within a 1.0 M hydrochloric acid (HCl) medium, leading to a remarkable usability exceeding 90% even after five consecutive adsorption/desorption cycles. Spike recovery experiments conducted using real water samples substantiate the practical applicability of the adsorbent. The high efficiency, utilization of cost-effective materials, and ease of fabrication, coupled with their selective nature and lower environmental impact through sorbent reuse, collectively confer superior advantages. These distinctive features render the environmentally benign adsorbent highly applicable for promising applications in the removal of methylene blue from aqueous solutions.

## 1 Introduction

Water-soluble organic dyes pose a significant environmental threat as they are discharged into rivers and sewage systems, leading to contamination of both natural habitats and drinking water sources.^1,2^ Given the potential toxicity of these dyes to human health, preemptive removal measures are imperative to safeguard the water ecosystem from their deleterious effects. Methylene blue (MB), a cationic dye, finds extensive use in printing, leather, and textile sectors, producing significant amounts of dye wastewater all over the world.^3,4^ Thus, efficient removal systems are of great importance. Several methods, viz., coagulation,^5^ photocatalytic degradation,^6^ biological treatment,^7^ membrane filtration,^8^ irradiation,^9^ and adsorption,^10−12^ were applied for the removal of MB from waters. Among these techniques, adsorption method is considered to be the most effective as it is rapid, selective, feasible, convenient, and simple.^13^ Up to now, a variety of adsorbents including clay, activated carbon, silica, and polymers have been investigated for the treatment of MB-contaminated waters.^14^ Recently, the application of polymeric materials as adsorbents has increased with developments in industrialization. Their widespread utilization in environmental remediation investigations is attributed to their exceptional mechanical durability, flexibility, and cost-effectiveness.^15,16^ Therefore, the synthesis and design of stable and selective polymeric adsorbents are still exciting.

It is crucial to create polymers with a variety of physical and chemical properties for use in the industry. The most significant of alternative ways to reach a sustainable improvement in the world is to produce environmentally friendly polymers since green, degradable, and/or recyclable polymeric materials are highly desirable as they provide a means for the minimization of the environmental pollution.^17−20^ Moreover, replacing traditional solvents and catalysts with appropriate alternatives for synthesis of new compounds has received widespread attention due to their environmental friendliness.^21−23^ With the proposed study, it was aimed to synthesize polymers that would minimize and eliminate the use of hazardous materials and methods that would cause harmful and toxic effects.

In this context, the monomer d-limonene (Lim), a colorless liquid produced as a byproduct of the orange juice industry’s extraction of orange peels, was used in the study. Lim is generated from more than 70 million kg of oranges that are produced worldwide each year in almost pure form (∼95%).^24^ It is frequently used as a flavoring ingredient in the medicinal, cosmetic, and food industries (hand cleaner, perfume, chewing gum, and ointment) because of its distinctive aroma.^25^ Lim, owing to its low toxicity and non-water-soluble properties, is increasingly employed as an environmentally friendly substitute for hazardous solvents like fluorinated compounds, toluene, xylene, methyl ethyl ketone, and chlorinated organic solvents in cleaning and separation applications.^26,27^ Moreover, in recent years, significant progress has been made in the synthesis and functionalization of Lim-based materials, paving the way for their utilization in environmental remediation, healthcare, and renewable energy applications. These materials exhibit unique properties, including high surface area, tunable porosity, and excellent adsorption capabilities, which render them highly suitable for addressing pressing societal and environmental challenges.^26,27^ The synthesis of d-limonene-based materials typically involves simple and cost-effective methods, such as polymerization, cross-linking, and surface modification, offering scalability and versatility for large-scale production. Moreover, the inherent chemical reactivity of d-limonene enables the incorporation of functional groups, allowing for tailored properties and enhanced performance in specific applications.^27^ In environmental remediation, d-limonene-based materials have demonstrated remarkable efficacy in the removal of pollutants forecasting high adsorption capacities, coupled with their low environmental footprint, that make them promising candidates for mitigating pollution and improving water and air quality.^28^ In the healthcare sector, d-limonene-based materials have shown potential for drug delivery, tissue engineering, and antimicrobial applications. Their biocompatibility, controlled release capabilities, and ability to mimic natural extracellular matrices make them attractive for biomedical applications, offering solutions for drug delivery systems and tissue regeneration therapies.^29^ In the realm of renewable energy, d-limonene-based materials hold promise for energy storage, catalysis, and solar cell technologies. Their tunable properties, stability, and compatibility with various electrolytes and substrates make them suitable for enhancing the efficiency and performance of energy storage devices and catalytic processes.^30^

In this study, divinylbenzene (DVB) served as a cross-linker, capitalizing on its suitability for generating porous polymeric materials with favorable mechanical properties through free radical polymerization.^31^ Moreover, DVB contributes to an increased specific surface area in adsorbents, facilitating effective dye molecule adsorption through π–π interactions between aromatic rings and dye molecules.^32^ To address this objective, a novel adsorbent, poly(LIM-*co*-DVB-*co*-AMPS), was meticulously designed, offering versatility, simplicity, and environmental friendliness for the removal of Methylene Blue (MB) from aqueous solutions. Following synthesis, comprehensive characterization using techniques such as Brunauer–Emmett–Teller (BET) surface area analysis, thermal gravimetric analysis (TGA), scanning electron microscopy (SEM), ζ potential measurement, Fourier transform infrared spectroscopy (FTIR), and elemental analysis confirmed the morphology and structure of the adsorbent. The efficacy of the adsorbent in removing MB from aqueous solutions was systematically investigated, considering key experimental parameters such as pH, contact time, and adsorbent dosage. The experimental data were rigorously analyzed by using the Dubinin–Radushkevich (D-R), Freundlich, and Langmuir isotherm models. Furthermore, the study delved into the kinetics of the process, employing intraparticle diffusion (ID), pseudo-first-order (PFO), and pseudo-second-order (PSO) models. To validate the real-world applicability, the proposed adsorbent was tested against samples of tap water, ultrapure water, bottled drinking water, and industrial wastewater to assess its removal effectiveness across diverse water matrices. This multifaceted approach ensures a comprehensive understanding of the adsorbent’s performance, paving the way for its potential application in water purification processes.

## 2 Experimental Section

### 2.1 Materials, Methods, and Apparatus

All of the substances and solvents utilized were analytical reagent quality. To create a stock solution of methylene blue (MB) at a concentration of 1000 mg/L, an appropriate amount of MB was dissolved in ultrapure water with a resistance of 18.2 MΩ. Lower concentration standards of MB were freshly prepared on a daily basis. D-limonene (LIM, Sigma-Aldrich), cross-linker divinylbenzene (DVB, Sigma-Aldrich), and 2-acrylamido-2-methyl-1-propanesulfonic acid (AMPS, Sigma-Aldrich) were utilized without further modification. The initiator 2,2′-azobis(isobutyronitrile) (AIBN, Merck) underwent purification through a series of consecutive crystallization steps employing a chloroform–methanol mixture (1:2, v/v).

UV–visible spectroscopy (UV–vis) (spectrophotometer, Cary 60, Agilent) was used for the determination of MB. The FTIR spectra of the synthesized adsorbent were obtained by using a PerkinElmer IR spectrometer. The thermal analysis of the adsorbent was conducted using a thermal gravimetric analysis/differential thermal analysis/differential thermogravimetric analysis simultaneous system (Hitachi 7000, TGA/DTA/DTG). The analysis was performed at a heating rate of 10 °C/min under a nitrogen atmosphere, spanning from room temperature to 600 °C. In order to examine the morphology of the adsorbent, a Thermo Scientific scanning electron microscope was employed. For assessing the textural properties, including pore volume, pore size, and Brunauer–Emmett–Teller (BET) surface area of the adsorbent, a Micromeritics ASAP 2020 surface area analyzer was used through N_2_ adsorption at −196 °C. The elemental composition of the adsorbent was determined through analysis with a LECO Truspec Micro CHSN analyzer. Batch type adsorption studies were conducted by using a water bath shaker (Nüve ST 402) equipped with a controlled thermostat. The pH measurements were performed using a Hanna pH meter with a suitable electrode. The determination of the point of zero charge (pH_*pzc*_) for the adsorbent was conducted utilizing the batch equilibration method.^33^ In this procedure, 50.0 mg of the sorbent was immersed in 20.0 mL of ultrapure water. The pH of the suspension was systematically adjusted from approximately 2.0 to around 10.0 using either 0.1 M HNO_3_ or NH_3_ solutions. The equilibration process was carried out for 12 h under controlled conditions in a thermostated shaker set to 25 °C. Following equilibration, the suspensions were filtered, and the equilibrium pH values were meticulously recorded. To ascertain the pH_*pzc*_, a plot of ΔpH (calculated as pH_final_ – pH_initial_) versus pH_initial_ was constructed, enabling the identification of the point at which these values intersected.

### 2.2 Synthesis of Poly(LIM-*co*-DVB-*co*-AMPS)

**Figure 1:**
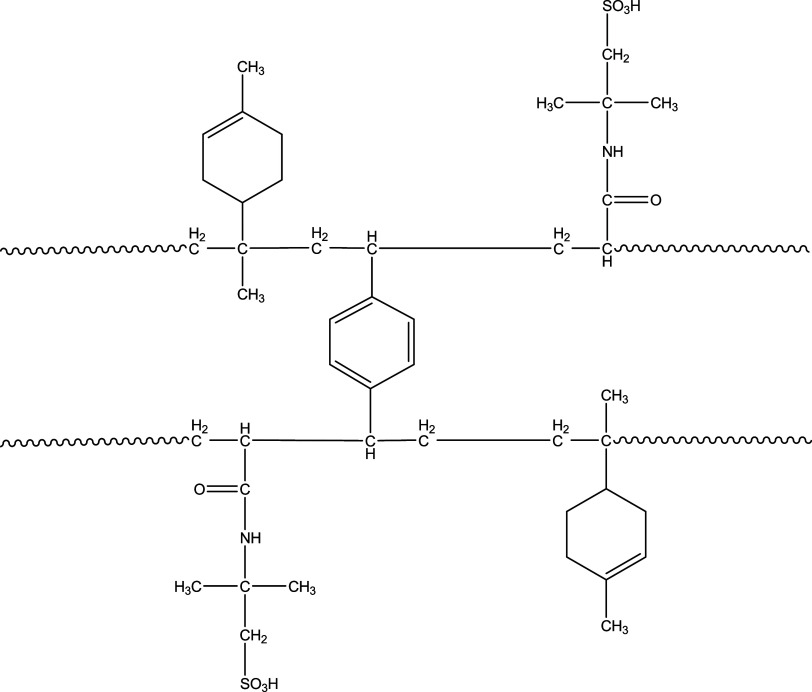
Chemical structure of the poly(LIM-*co*-DVB-*co*-AMPS).

The synthesis of the adsorbent was conducted in the laboratory using a radical initiator (AIBN) in a dimethylformamide solution. During the synthesis, a polymerization flask was used to combine two suitable monomers, namely, LIM (1.2 mL) and AMPS (1.0 g), along with 1.1 mL of DVB and 0.025 g of AIBN. This mixture was kept under a nitrogen atmosphere for 3 h at a temperature of 70 °C. After this step, the resulting adsorbent was filtered, washed with diethyl ether, and then vacuum-dried at 50 °C until a consistent weight was attained. As a result, poly(LIM-*co*-DVB-*co*-AMPS) was successfully synthesized (Figure 1).

### 2.3 Sorption/Desorption Experiments

Adsorption experiments were conducted in batch mode using 50.0 mL falcon tubes by varying several experimental conditions including pH values from 2.0 to 10.0, contact times ranging from 1.0 to 60.0 min, and adsorbent dosages from 0.2 to 1.0 g L^–1^ in a thermostatic water bath at 25 °C. The liquid and solid phases were separated through filtration using PTFE filters, and the amount of MB in the filtrate was quantified using UV–vis spectroscopy.

Desorption experiments were performed using several eluents (HNO_3_, HCl, H_2_SO_4_, CH_3_COOH, ethanol, and methanol). Hence, the adsorption studies were first conducted using ideal conditions, where 10.0 mL of eluent was introduced to the adsorbent containing MB. The mixture was then shaken for 60.0 min, and the concentration of MB in the resulting solution was analyzed using UV–vis spectroscopy.

### 2.4 Adsorption Isotherms and Kinetics

The equilibrium relationship between adsorbent and adsorbate was studied through the application of adsorption isotherms that also give the capacity of the adsorbent. It is also an essential tool in describing the adsorption phenomena occurring at different types of interfaces. In this research, the suitability of three different models, viz., Dubinin–Radushkevich (D-R), Freundlich, and Langmuir, was examined for fitting the experimental data and describe the adsorption process effectively.

A kinetic analysis was also carried out to gain insights into the rate of removal of the metal ions from the solutions since it offers valuable insights into identifying the step that controls the rate and the mechanism governing the adsorption reaction. Three well-established kinetic models, specifically the pseudo-first-order (PFO), pseudo-second-order (PSO), and intraparticle diffusion (ID) models, were evaluated to investigate and predict the reaction kinetics.

## 3 Results and Discussion

### 3.1 Characterization

**Figure 2:**
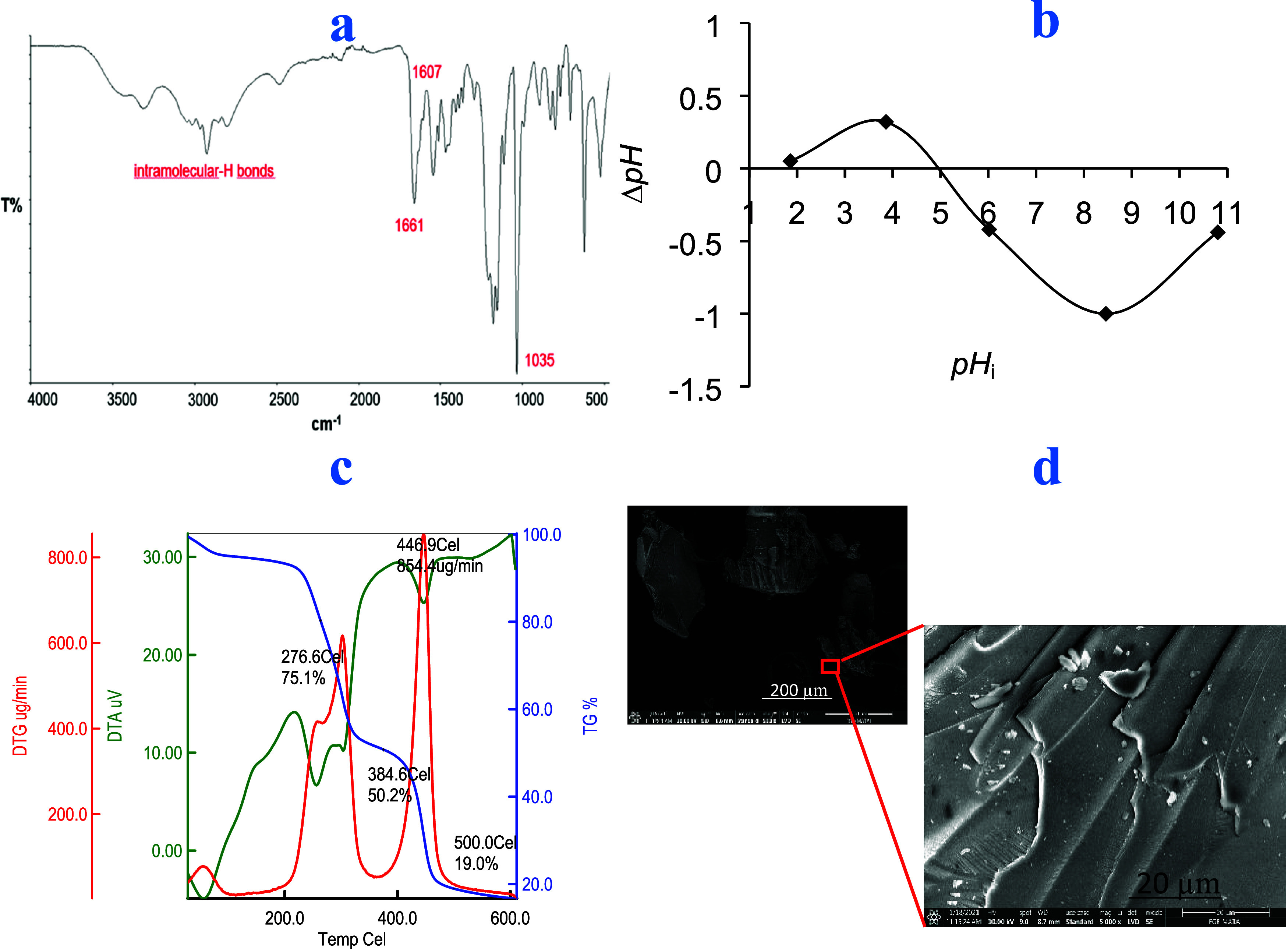
(a) FTIR spectra, (b) ζ potential, (c) thermal profile, and (d) SEM image of the polymer

The characterization of the adsorbent involved a systematic analysis and assessment of its physical, chemical, and structural properties. Utilizing Fourier transform infrared spectroscopy (FTIR), as illustrated in Figure 2a, facilitated the identification of distinct functional groups within the polymer matrix. Notably, prominent absorption bands were discerned within the spectrum, notably a band ranging from 3500 to 3200 cm^–1^ indicative of intramolecular -H bonding. Additionally, stretching vibrations were evident at specific wavenumbers: 1661 cm^–1^ corresponding to HN-C=O, 1607 cm^–1^ associated with C=C bonds within the aromatic ring, 3016 cm^–1^ representing CH stretching in aromatic structures, and 1035 cm^–1^ attributed to S–O stretching in the adsorbent material.

The thermal stability assessment of the adsorbent underwent meticulous scrutiny by utilizing a TGA/DTA/DTG simultaneous system, which yielded valuable insights into its thermal behavior. The thermogram unveiled a dual-stage degradation process, indicating discernible levels of decomposition within the adsorbent matrix. Noteworthy decompositions were delineated by distinct temperatures: 264 °C marked 20% decomposition, 277 °C denoted 25% decomposition, and 386 °C indicated 50% decomposition. Moreover, progressive weight losses were observed, comprising 51% at 400 °C, 71% at 450 °C, and 81% at 500 °C, with residual amounts remaining at 550 °C (18%) and 600 °C (17%). The maximal decomposition temperature recorded stood at 447 °C. The thermal curves detailing the adsorbent’s behavior are shown in Figure 2c. This thermal analysis significantly enriches our comprehension of the material’s resilience across varying temperature gradients, a pivotal consideration for its prospective deployment across diverse environmental contexts.

The analysis of pore volume, BET surface area, and average pore diameter involved N2 adsorption after degassing at 80 °C. The nitrogen adsorption–desorption isotherm data suggested that the material exhibited a Type-III adsorption isotherm behavior, as indicated by the hysteresis loop observed in the P/Po range of 0.4–1.0. The type of isotherm behavior exhibited by the polymer suggested a porous structure, indicating that adsorption predominantly occurred on the external surface or within large pores rather than within the internal structure (Figure S1). Furthermore, the BET specific surface area is determined to be 2.3 m^2^ g^–1^, with an average pore size of 3.15 nm and a total pore volume of 0.0036 cm^3^g^–1^, underscoring the nature of the adsorbent, which is crucial for facilitating adsorption processes. Notably, the presence of pores larger than 2 nm is anticipated to promote rapid accessibility between MB molecules and the functional groups within the adsorbent matrix, as elucidated by previous studies.^34^ Moreover, the determination of the adsorbent’s point of zero charge (pH_*pzc*_) through batch equilibration yielded a value of 5.20 (Figure 2b). This parameter is fundamental in understanding the surface charge characteristics of the adsorbent material, influencing its interaction with charged species, such as MB dye molecules in solution. This value indicated the equilibrium point where the surface possesses neither a net positive nor a negative charge, offering insights into the adsorption behavior under varying pH conditions. Additionally, elemental analysis revealed the elemental composition of the adsorbent, with nitrogen (N) comprising 5.75%, sulfur (S) 1.65%, hydrogen (H) 7.25%, and carbon (C) 61.3%. This elemental composition provides further insight into the chemical makeup of the adsorbent, aiding in understanding its potential interactions with target molecules and its suitability for specific adsorption applications.

Scanning electron microscopy (SEM) served as an instrumental technique for the detailed examination of morphological characteristics and surface attributes of the sorbent material. The scanning electron micrographs, depicted in Figure 2d, were acquired at magnifications of 500× and 5000×, allowing for comprehensive visualization. Insights gleaned from these images unveil a surface morphology characterized by nonuniformity and a rough topography, indicative of the sorbent’s complex structure. Additionally, the porous nature of the material is distinctly evident, manifesting in the form of internal fissures and microscopic voids interspersed throughout. It is postulated that the nonhomogeneous topographical features observed in the sorbent material hold significant implications for its adsorption properties. Specifically, the presence of such features is anticipated to result in an expanded surface area, thereby augmenting the adsorption capacity for MB and other target molecules. The surface area, coupled with the porous structure, creates an environment boosting the efficacy of the sorbent in pollutant removal applications and environmental remediation studies.

### 3.2 Influence of pH

pH is a highly significant parameter in adsorption studies since it affects the interaction between the substances in the solution, specifically involving hydroxyl/hydrogen ions and the active sites of the adsorbents. Researchers, engineers, and scientists need to carefully consider and control pH to ensure effective and efficient sorption in a wide range of applications.^35^ Hence, it is imperative to explore the influence of the pH on the adsorption of MB by the adsorbent. For this purpose, 10.0 mg of the adsorbent was added to a 10 mL solution containing 10.0 mg L^–1^ of MB. The mixture was shaken for 12 h at a constant temperature of 25 °C while varying the pH values within the range of 2.0 to 10.0. The mixture was subsequently separated through filtration, and the concentration of MB was quantified by using UV–vis spectroscopy. The results indicated that effective MB adsorption occurred within the pH range of 6.0 to 10.0. Notably, the dye adsorption exhibited its highest removal rates in neutral and alkaline environments, while the removal efficiency in acidic conditions was comparatively lower. At a pH of 4, the efficacy of dye removal was quantified at 75%, yet it exhibited an increase with rising pH levels. This occurrence can be elucidated by the positively charged state of the MB dye within the solution, rendering it more prone to interacting with substrates possessing a net negative charge. Upon reaching a pH of 6, the removal efficiency approached nearly 99%. Under alkaline conditions, this can be ascribed to the disassociation of sulfonic acid groups, resulting in the formation of stable ionic groups that improve electrostatic attraction or ionic bonding between MB and the adsorption sites on the polymer.^36^ Conversely, in acidic environments, protonation of the amide groups of AMPS ensued, engendering repulsive electrostatic forces between the adsorption sites on the polymer and the MB molecules, thereby diminishing the removal of the dye molecules.^37^

**Figure 3:**
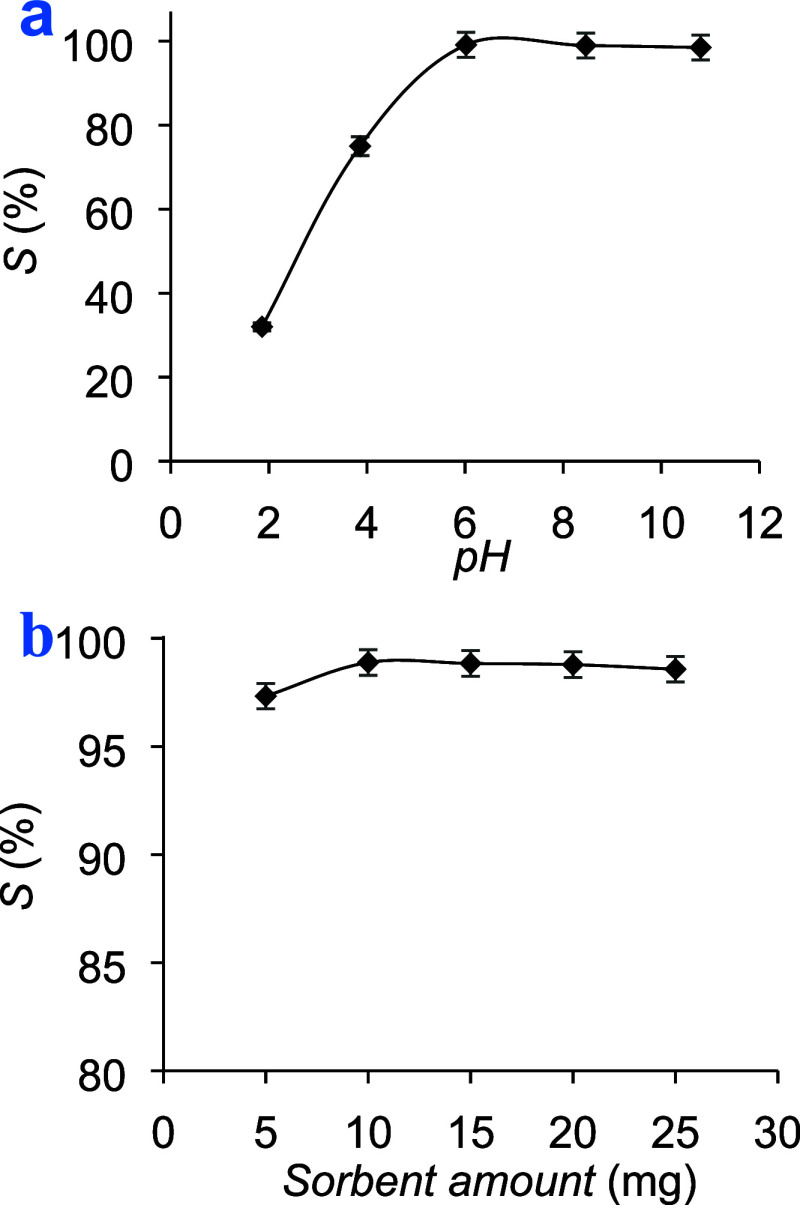
Influence of (a) pH and (b) sorbent dose on the adsorption of MB using a polymer.

This observation aligns with the concept of the point of zero charge of the adsorbent, which highlights the significance of the pH_*pzc*_ in determining the surface charge of the adsorbent. The pH*_pzc_* of the adsorbent determined by batch equilibration method was found to be 5.2.^38^ Below the pH_*pzc*_ value of 5.2, the surface charge remained positive, while it became negative above the pH_*pzc*_ value. This observation suggested that when the solution’s pH is equal to or less than 5.2, the polymer surface contains acidic groups, attracting excess hydrogen ions (H^+^), thereby yielding a positive potential within an acidic medium. Conversely, as the pH increased, the polymer surface had a greater number of hydroxide ions, resulting in a negatively charged surface potential. Consequently, with increasing solution pH, the adsorbent surface is assumed to have a negative charge, facilitating the adsorption of cationic dye molecules, attributed to the electrostatic attraction between the negatively charged surface and the positively charged dye cations (Figure 3a).

### 3.3 Influence of Adsorbent Dosage

Adsorbent dose is also a critical parameter in sorption studies that span a broad spectrum of applications from environmental remediation to industrial processes. Understanding the significance of the quantity of adsorbents used is essential for assessing the effectiveness and feasibility of sorption-based technologies. The selection of the optimal adsorbent dosage must be conducted with care in order to obtain higher adsorption rates and large adsorption capacity.^39^ The optimization study was conducted by varying the adsorbent doses from 0.2 to 1.0 g adsorbent per liter while maintaining a constant concentration of 10.0 mg L^–1^ of MB, a contact time of 30.0 min, and a pH of 7.0. The analysis of methylene blue (MB) adsorption as a function of sorbent quantity yielded insightful observations. Upon examination of the data presented in Figure 3b, it becomes apparent that the percentage of MB adsorption does not exhibit a significant trend with increasing sorbent quantity. Across the range of sorbent amounts tested, which ranged from 5 to 25 mg, the percentage of MB adsorption remained relatively stable. Specifically, the adsorption percentages ranged from 97.3 to 98.9% for the different sorbent quantities, showcasing consistent levels of MB removal regardless of the amount of sorbent utilized. Consequently, for all subsequent experiments, an adsorbent dose of 0.4 g L^–1^ was utilized.

### 3.4 Influence of Contact Time and Adsorption Kinetics

Contact time is a fundamental parameter in sorption studies. It enables the assessment of adsorption kinetics, system optimization, and attainment of equilibrium. Understanding the role of contact time is essential for designing effective sorption systems in various applications and gaining insights into the underlying mechanisms of sorption processes. The efficiency and the removal rate of the adsorbents are maximized with the use of appropriate contact times.^40^ For this reason, the adsorption experiments are realized under the conditions, viz., 10.0 mg L^–1^ MB, 0.4 g L^–1^ adsorbent dose, and pH of 7.0 for varying contact times from 1.0 to 60.0 min at 25 °C. The findings suggest that the adsorption of MB has increased with an extended contact time and after 30.0 min. This increase in adsorption efficiency can be attributed to the higher number of surface sites and good surface area. After 30 min, the adsorption of MB remained constant and no further increase in adsorption efficiency was observed as the adsorbent became saturated and no vacant adsorption sites are available. As a result, the optimum contact time for MB adsorption was set as 30.0 min and used in subsequent studies.

**Figure 4:**
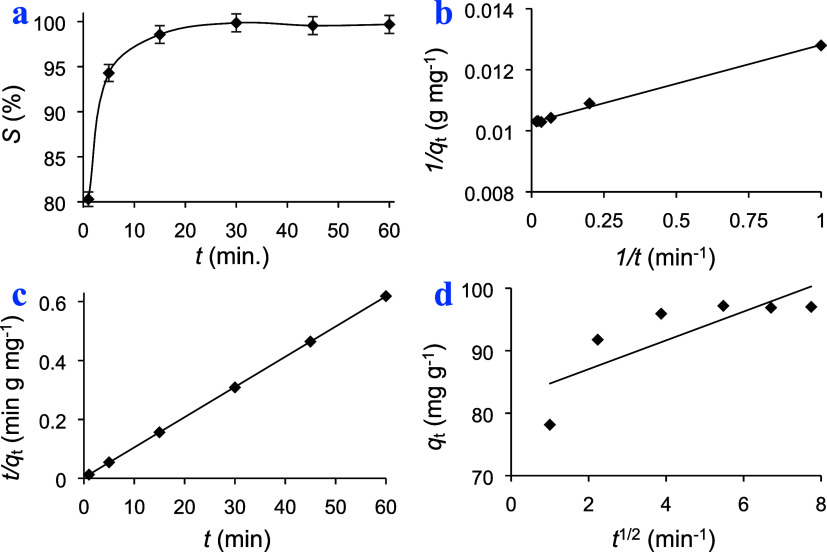
(a) Effect of contact time on the adsorption of MB using a polymer. (b) Pseudo-first order, (c) pseudo-second order, and (d) intraparticle diffusion models’ plots.

Under the optimized conditions, adsorption kinetics was investigated utilizing the equations stated previously.^41^ The adsorption kinetics was analyzed using two models, namely, pseudo-first-order (PFO) and pseudo-second-order (PSO). In addition to these models, the diffusion mechanism of the adsorption process was tested using an intraparticle diffusion (ID) model. Table 1 and Figure 4 indicate the fitting parameters, correlation coefficients (*R*^2^), and the equations of the aforementioned models. Based on the obtained results, the pseudo-second-order (PSO) model emerges as the most suitable framework for elucidating the adsorption kinetics of methylene blue onto the adsorbent. The exceptional fitting performance of the PSO model, reflected by a high correlation coefficient (*R*^2^ = 1), underscores its efficacy in capturing the intricate kinetics of MB adsorption. Moreover, the calculated equilibrium capacity of 97.1 mg g^–1^ closely mirrors the experimental capacity of 97.3 mg g^–1^, providing compelling evidence of the predictive power and reliability of the PSO model. These findings suggest that MB molecules interact with active sites on the adsorbent surface. The observed conformity between the PSO model predictions and experimental data signifies a controlled and systematic adsorption process. The intraparticle diffusion (ID) model provides valuable insights into the mechanism governing the transport of methylene blue (MB) molecules within the adsorbent matrix. Despite the moderate fitting performance of the ID model, as indicated by a correlation coefficient (*R*^2^) of 0.6489, intraparticle diffusion might contribute to the overall adsorption process, and it may not be the sole rate-limiting step. The calculated diffusion coefficient (kp) of 2.30 mg g^–1^ min^–1^/^2^ reflects the rate at which MB molecules penetrate the internal pores of the adsorbent. The presence of intraparticle diffusion highlights the importance of mass transfer phenomena in facilitating the transport of MB from the bulk solution to the adsorbent surface. However, the deviation from ideal behavior, as evidenced by the discrepancy between experimental and model-predicted capacities, implies the influence of additional factors, such as external mass transfer resistance or surface heterogeneity. Therefore, while intraparticle diffusion plays a discernible role in the adsorption process, its contribution may be modulated by other concurrent mechanisms. In summary, the consistent agreement can be concluded that the PSO model accurately depicts the dynamic adsorption behavior of MB onto the adsorbent.

**Table 1:** Kinetic Parameters of Various Models Fitted to the Experimental Data for the Adsorption of MB by a Polymer

kinetic model	equation	parameter	value
pseudo-first order		*R*^2^	0.9961
		*q*_1_ (mg g^–1^)	97
		*k*_1_ (min^–1^)	0.24
pseudo-second order		*R*^2^	1
		*q*_2_ (mg g^–1^)	97.1
		*k*_2_ (g mg^–1^ min^–1^)	0.04
intraparticle diffusion		*R*^2^	0.6489
		*C* (mg g^–1^)	82.4
		*k*_p_ (mg g^–1^ min^–1/2^)	2.30
experimental capacity		*q*_exp._ (mg g^–1^)	97.3

### 3.5 Adsorption Capacity and Isotherms

Adsorption capacity and isotherms are fundamental concepts in the field of adsorption science, playing a crucial role in understanding, quantifying, and optimizing adsorption processes. These concepts are of paramount importance in a wide range of applications spanning from environmental cleanup to the fields of chemical engineering and materials science. As a result, the adsorption capacity of the adsorbent was determined by exploring the relationship between the equilibrium concentration of the substance being adsorbed (in mg L^–1^) and the mass of the substance per unit mass of the adsorbent (in mg g^–1^). It was noted that the amount of MB adsorbed (*q*_e_) increased with the increase of initial MB concentration, ultimately reaching a maximum value of 97.3 mg g^–1^.

**Figure 5:**
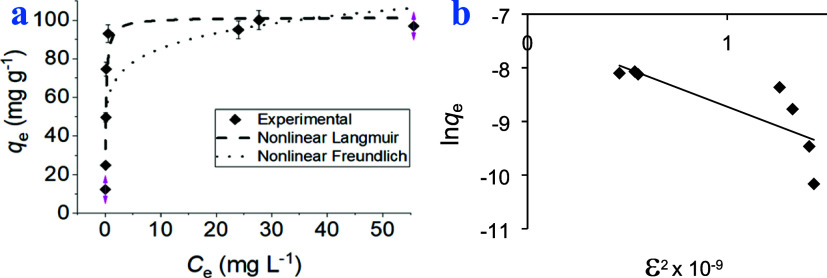
(a) Nonlinear fit of Langmuir and Freundlich isotherm models of MB adsorption onto the polymer with respect to experimental data and (b) Dubinin–Radushkevich model’s plot (*q*_e_ = mol g^–1^; *C*_e_ = mol L^–1^).

Under the equilibrium conditions, the design of the adsorption process was conducted with mathematical descriptions based on adsorption, called isotherms. The interpretation of the adsorption process involved the application of three isotherm models: Freundlich, Langmuir, and Dubinin–Radushkevich. The relevant equations can be found in a prior work.^41^ Both linear and nonlinear Langmuir and Freundlich models were applied for the adsorption of MB onto the polymer. The maximum adsorption capacity (*q*_m_) obtained from the Langmuir model was 98 mg g^–1^ for the linear case and 101.4 mg g^–1^ for the nonlinear case. The Langmuir model’s parameter, particularly the maximum adsorption capacity (*q*_m_), exhibits more consistency and proximity to the experimental value (*q*_exp_), suggesting a strong fit with the adsorption system, indicating a monolayer adsorption on a homogeneous surface. In contrast, the Freundlich and D-R models deviate notably from the experimental value, indicating a less precise fit to the data. Thus, the Langmuir model emerges as the most suitable model for describing the adsorption behavior of MB onto the polymer in this study, owing to its consistency and closeness to the experimental value (Table 2 and Figure 5).

**Table 2:** Parameters of Langmuir, Freundlich, and Dubinin–Radushkevich Sorption Isotherm Models for the Adsorption of MB by a Polymer

			value	
sorption model	equation	parameter	linear	nonlinear
Langmuir		*R*^2^	0.9996	0.8678
		*q*_m_ (mg g^–1^)	98	101.4
		*L* (L mg^–1^)	6.0	6.6
Freundlich		*R*^2^	0.5222	0.6079
		*K*_F_ ((mg g^–1^)(L mg^–1^)^1/n^)	54.1	63.5
		*n*	5	7.80
Dubinin–Radushkevich		*R*^2^	0.6347	
		*k* (mol^2^ J^–2^)	1.14 × 10^–9^	
		*X*_m_ (mol g^–1^)	0.67 × 10^–3^	
		*E* (kJ mol^–1^)	20.9	
experimental capacity		*q*_exp._ (mg g^–1^)	97.3	

### 3.6 Thermodynamics of Adsorption

Temperature, being one of the key factors in assessing the viability of the adsorption process, directly influences both the adsorption capacity and the rate at which the adsorbate is taken up. By carefully studying the temperature dependence of dye adsorption, it is possible to create treatment processes that are not only efficient but also cost-effective for eliminating dyes from industrial effluents and water resources.^42^ The relationships between Gibbs free energy change (Δ*G*°), enthalpy change (Δ*H*°), and entropy change (Δ*S°*) during the adsorption process offered valuable insights into the thermodynamic aspects of adsorption. Table 3 provides data at three different temperatures (298, 318, and 338 K). The negative values of Δ*G*° indicated that the adsorption of MB onto the polymer was a spontaneous process at all temperatures considered. This suggested that the adsorption occurs spontaneously and is thermodynamically favorable. The decrease in Δ*G*° values with increasing temperature suggested that the spontaneity of the adsorption process decreased slightly with increasing temperature. The negative values of Δ*S*° suggested that the adsorption process leads to a decrease in the disorder or randomness of the system. This is typical for adsorption processes, where the adsorbate molecules become more ordered upon adsorption onto the surface of the adsorbent. The decrease in magnitude of Δ*S*° values with increasing temperature suggested that the system becomes slightly more ordered at higher temperatures. The negative values of Δ*H*° indicated that the adsorption process was exothermic, meaning that heat was released during the adsorption of MB onto the polymer. The decrease in the magnitude of Δ*H*° values with increasing temperature suggested that the exothermic nature of the adsorption process becomes slightly weaker at higher temperatures.

**Table 3:** Thermodynamic Parameters for the Adsorption of MB by a Polymer

*T* (K)	Δ*G*° (kJ mol^–1^)	Δ*S*° (kJ mol^–1^ K^–1^)	Δ*H*° (kJ mol^–1^)
298	–29.1	–0.012	–32.8
318	–30.1	–0.009	
338	–30.3	–0.007	

### 3.7 Desorption, Repeated Use, Method Performance, and Selectivity of the Adsorbent

Desorption process is very important due to the economic and effective use of the adsorbents, especially in practical applications. A suitable eluent should effectively remove the dye without causing damage to the structure and functional groups of the adsorbent. Initially, several acids (HNO_3_, HCl, H_2_SO_4_, and CH_3_COOH) at various concentrations (0.01, 0.1, and 1.0 M) and different kinds of organic solutions (ethanol, methanol, and acetonitrile) were tried. The results indicated that the desorption ability of the aforementioned eluents ranged only between 8 and 70%. According to the findings of Greluk and Hubicki,^43^ desorption process can notably be improved by applying a mixture of acid and alcohol. Thus, mixtures of acid and organic solutions (1.0 M acid in organic solution) were tried, and among the studied eluents, 1.0 M HCl in ethanol exhibited the most effective dye desorption efficiency (Supplementary File T1). As a result, subsequent dye desorption experiments were carried out using this eluent.

Reusability is also a significant factor that should be studied to minimize the environmental impact of the method and understand the performance of the sorbent. In the view of this, the reusability of the adsorbent was examined by five consecutive cycles of adsorption/desorption steps. The results indicated a little decrease in the sorption/desorption efficiency of the adsorbent, but no significant loss (<10%) was observed even after five cycles (Figure S2). These evaluations showed that the adsorbent is stable and can readily be used in practical applications.

In order to evaluate the method’s performance, spike recovery experiments were conducted with samples of tap water, industrial waste, bottled drinking water, and ultrapure water. Appropriate amounts (1.0, 5.0, and 10.0 mg L^–1^) of MB were spiked into the aforementioned water samples, and the proposed method (adsorption/desorption) was utilized with the optimum conditions (pH of 7, adsorbent amount of 10.0 mg, and contact time of 30.0 min). Table 4 shows the findings, demonstrating that all water types achieved satisfactory recovery values (>90%) with low relative standard deviation values within the range of 0.2–1.5%. The high recovery values gathered in the study indicate the applicability of the method. As a result, it can be affirmed that the adsorbent exhibits significant promise as a material for effectively removing MB in analytical applications.

**Table 4:** Spike Recovery Results of MB in Real Samples (n = 3)

sample	added (mg L^–1^)	sorption (%)	recovery (%)
ultrapure water	1.0	99.1 ± 0.2	99.6 ± 0.3
	5.0	98.2 ± 0.2	98.7 ± 0.2
	10.0	96.4 ± 0.3	99.1 ± 0.2
bottled drinking water	1.0	97.1 ± 0.2	97.9 ± 0.2
	5.0	98.8 ± 0.6	98.7 ± 0.3
	10.0	95.5 ± 0.4	99.2 ± 0.2
tap water	1.0	98.3 ± 0.4	98.7 ± 0.2
	5.0	96.2 ± 0.2	98.5 ± 0.3
	10.0	99.5 ± 0.5	99.1 ± 0.4
industrial wastewater	1.0	97.2 ± 0.4	99.2 ± 0.3
	5.0	98.1 ± 0.8	98.8 ± 0.6
	10.0	96.6 ± 1.5	97.7 ± 0.5

Selectivity is also crucial as it determines the ability of a material to preferentially adsorb a target molecule from a mixture, enabling efficient separation and purification processes in various applications such as environmental remediation, wastewater treatment, and chemical synthesis. Thus, the evaluation of the selectivity of our experimental materials for methylene blue involved a systematic investigation employing a range of analytical techniques. Initially, batch adsorption experiments were conducted, wherein methylene blue was subjected to adsorption alongside a selection of model dyes commonly encountered in aqueous solutions, including methyl orange, allura red, brilliant blue, malachite green, sunset yellow, and tartrazine. Through the analysis of the percent adsorption values obtained for methylene blue and the comparative model dyes, we delineated the relative affinity of our material toward methylene blue. Thus, it was concluded that our adsorbent demonstrated notable selectivity for MB, highlighting its potential efficacy in targeted removal applications from aqueous solutions (Figure S3).

### 3.8 Comparison with Other Methods

Table 5 presents a thorough comparison between the findings of the current study and those reported in the literature, focusing on key parameters such as pH, contact time, adsorption capacity, temperature, isotherm, and kinetic models.^44−51^ The comparison highlights significant variations and contributes to a comprehensive understanding of the proposed method’s efficacy in comparison to existing approaches. The table highlights significant disparities in adsorption capacity values across various adsorbents prepared under diverse conditions. The observed variations in adsorption capacity values among different adsorbents prepared under varying conditions, as indicated in the table, led to the conclusion that the proposed adsorbent exhibits a noteworthy adsorption capacity for the effective removal of MB from aqueous solutions. The synthesis of the polymer, with an easy laboratory setup using standard laboratory equipment under mild reaction conditions (inexpensive, easy to find and green reagents, mild reaction temperature, and atmospheric pressure), constitutes a distinctive feature of the proposed adsorbent. These features distinguish favorably when compared with other methods reported in the literature.

**Table 5:** Comparative Analysis of Proposed and Published Methods for MB Removal

sorbent	pH	adsorbent dosage (g)	contact time (min)	temperature (°C)	capacity (mg g^–1^)	isotherm model	kinetic study	reference
magnetic boehmite composite	7	0.05	180	25	70.03	Langmuir	PSO	(44)
cyclodextrin-modified magnetic nanospheres	7	0.005	40	25	305.8	Langmuir	PSO	(45)
chitosan-zeolite zwitterion composite	9	0.2	180	30	156.1	Freundlich	PSO	(46)
sugar cane bagasse biochar	7.4	0.03	180	30	38.76	Langmuir	PSO	(47)
granular aerobic sludge	6	0.25	60	25	381.7	Langmuir	PSO	(48)
phragmites waste	7	0.2	150	4	54.9	Both	PSO	(49)
low cost activated sludge		0.06	10	25	366.3	Langmuir	PSO	(50)
modified-activated sludge composite	6	0.02		25	181.4	Both	PSO	(51)
poly(LIM-*co*-DVB-*co*-AMPS)	7	0.01	30	25	98	Langmuir	PSO	this work

## 4 Conclusions

A novel, convenient, cost-effective, highly efficient, and green polymer was created and applied for removing MB from aqueous solutions. The characterization studies performed using FTIR, SEM, TGA/DTA/DTG, BET, elemental, and ζ potential techniques confirmed that the polymer was successfully synthesized. The optimal conditions of the adsorption process were evaluated as follows: pH of 7.0, contact time of 30.0 min, and adsorbent dose of 0.4 mg L^–1^ at 25 °C. The experimental data exhibited an excellent fit with the Langmuir isotherm model, indicating a uniform distribution of methylene blue on the active sites of the adsorbent. The kinetic investigation of the experimental data specified that the adsorption process is more accurately correlated with the PSO model than PFO and ID models. The reusability experiments using ethanol in 1.0 M HCl as the eluent have shown that the adsorbent could be used repeatedly since less than 10% loss in the adsorption behavior of the adsorbent was observed even after five adsorption/desorption cycles. The considerable adsorption selectivity of the adsorbent ensured the possibility of separating MB even in the presence of various metal ions. The performance of the method was assessed with spike recovery studies involving real water samples, and the accurate results obtained confirmed the applicability of the polymer in various types of matrices. Finally, the proposed polymer is suggested as a green, feasible, efficient, and selective adsorbent with superior advantages of low cost, being environmentally benign, and ease of fabrication especially in ecological applications.

## Supporting Information Available

N_2_ adsorption–desorption isotherm of the polymer, efficiency of various eluents for the desorption of MB, reusability of the adsorbent, selectivity for methylene blue and model dyes (PDF)
