# Supplementary material for: Greener Synthesis of Poly(LIM-co-DVB-co-AMPS): A Sustainable Approach to Methylene Blue Removal
Source: ACS Omega. 2024 Dec 12;9(51):50147–57. doi: 10.1021/acsomega.4c00354 (PMC11683600; doi:10.1021/acsomega.4c00354)
Supplement: Supplementary file 1 — ao4c00354_si_001.pdf [file ao4c00354_si_001.pdf]

Greener Synthesis of poly(LIM-co-DVB-co-AMPS): A Sustainable Approach to Methylene Blue Removal

Aslı Erdem Yayayürük[a], Nevin Çankaya[b], Onur Yayayürük[a]*

[a]Ege University, Faculty of Science, Department of Chemistry, İzmir, 35100, Turkey

[b]Uşak University, Vocational School of Health Services-Oral and Dental Health Department, Uşak, 64200, Turkey

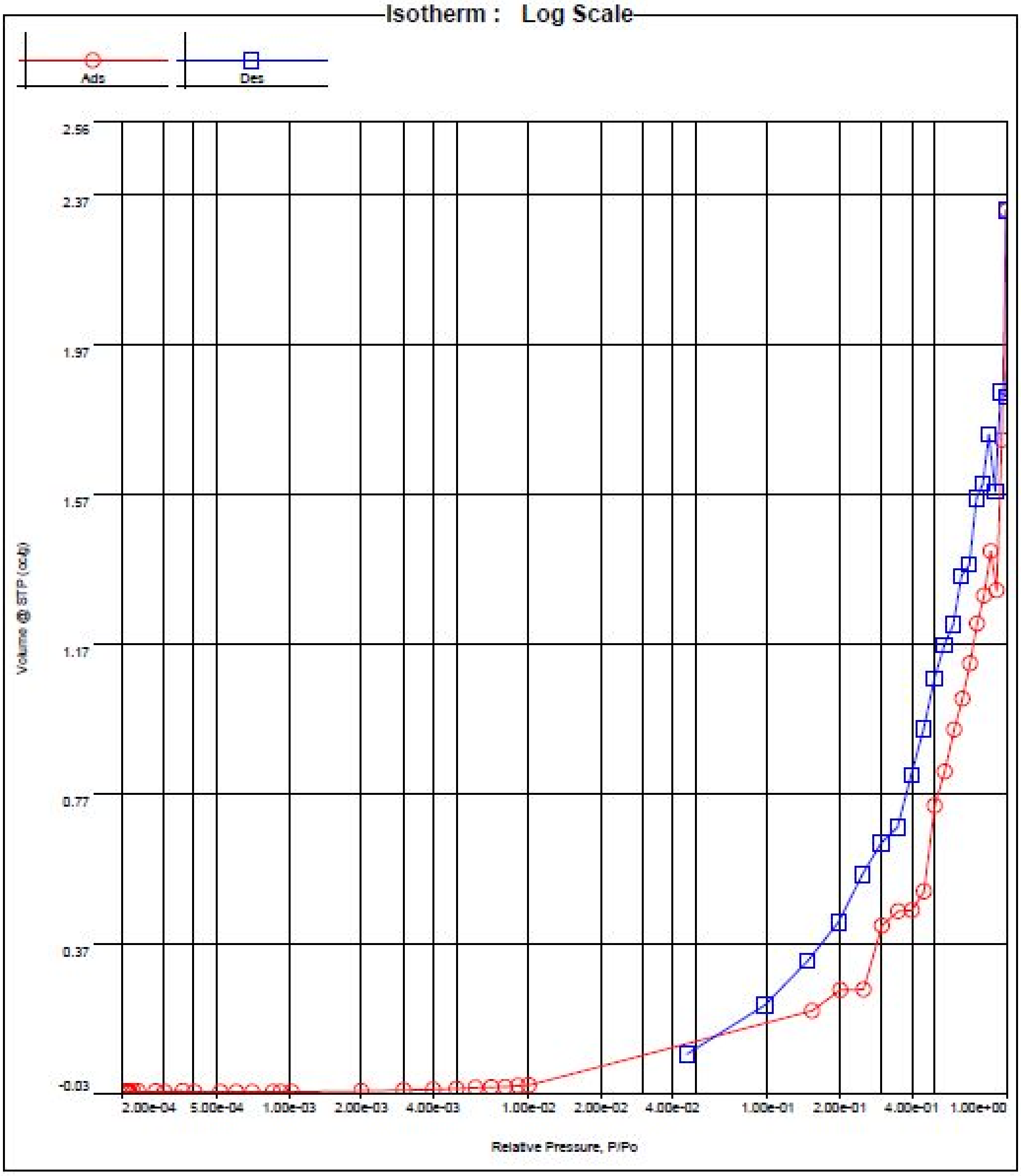

Figure S1. N_2 adsorption-desorption isotherm of poly(LIM-co-DVB-co-AMPS).

Eluent		Elution %
HNO_3	0.01 M	45.8
	0.1 M	56.9
	1.0 M	55. 2
HCl	0.01 M	20.8
	0.1 M	50.7
	1.0 M	70.3
H_2SO_4	0.01 M	27.1
	0.1 M	30.2
	1.0 M	25.4
CH_3COOH	0.01 M	8.3
	0.1 M	11.2
	1.0 M	17.8
Ethanol		12.7
Methanol		11.2
Acetontril		22.8
1.0 M HCl in ethanol		99.6

Table S1. Efficiency of various eluents for the desorption of MB from the adsorbent.

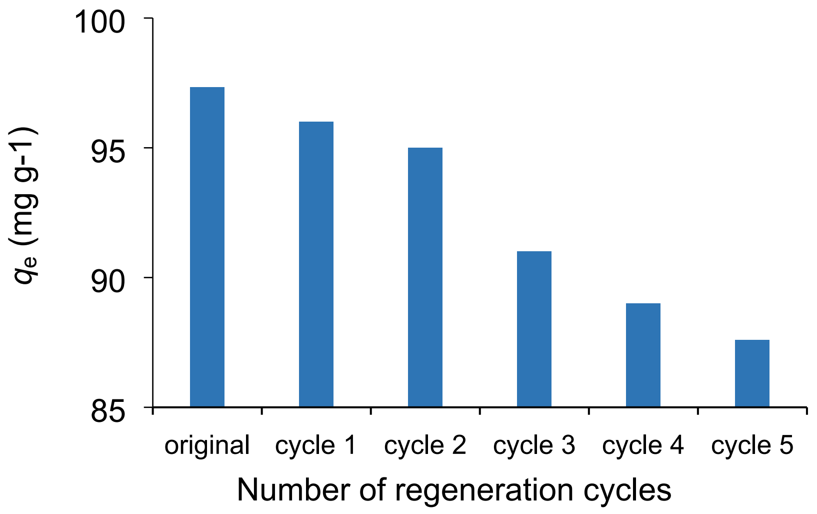

Figure S2. Reusability of the adsorbent over consecutive adsorption/desorption cycles

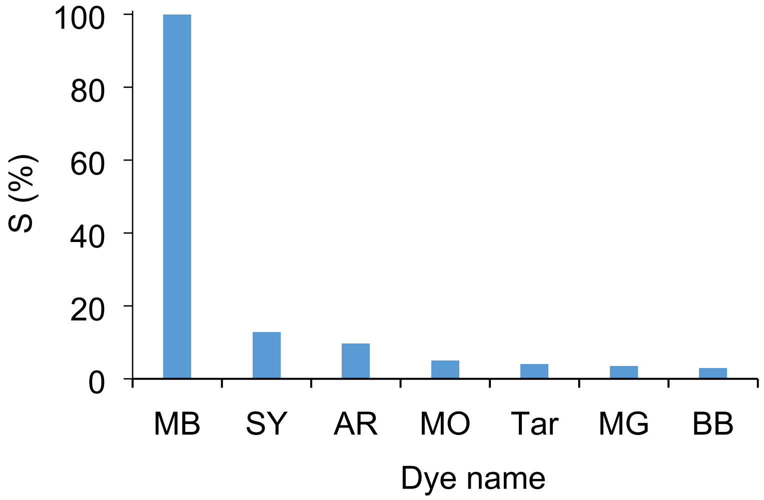

Figure S3. Evaluation of selectivity for methylene blue and model dyes (methyl orange (MO), allura red (AR), brilliant blue (BB), malachite green (MG), sunset yellow (SY), and tartrazine (TAR)) in aqueous solutions.
